# Masitinib in the treatment of active rheumatoid arthritis: results of a multicentre, open-label, dose-ranging, phase 2a study

**DOI:** 10.1186/ar2740

**Published:** 2009-06-23

**Authors:** Jacques Tebib, Xavier Mariette, Pierre Bourgeois, René-Marc Flipo, Philippe Gaudin, Xavier Le Loët, Paul Gineste, Laurent Guy, Colin D Mansfield, Alain Moussy, Patrice Dubreuil, Olivier Hermine, Jean Sibilia

**Affiliations:** 1 Service de Rhumatologie, Centre Hospitalier Lyon-Sud, 765 chemin du Grand-Revoyet, 69495 Pierre-Bénite, France; 2 Service de Rhumatologie, Hôpital Bicêtre, Assistance Publique-Hôpitaux de Paris, 78 rue du Général Leclerc, 94275 Le Kremlin-Bicêtre Cedex, France; 3 Service de Rhumatologie, Groupe Hospitalier Pitié Salpétrière, 83 bd de l'Hôpital, 75013 Paris, France; 4 Service de Rhumatologie, Hôpital Roger Salengro, Rue du Professeur Emile Laine, 59037 Lille cedex, France; 5 Service de Rhumatologie, CHU Hôpital Sud – GREPI-TIMC-IMAG UMR CNRS 5525, Avenue de Kimberley, 38434 Échirolles, France; 6 Service de Rhumatologie, CHU Hôpitaux de Rouen, 1 rue de Germont, 76230 Rouen, France; 7 AB Science, S.A., 3 avenue Georges V, 75008 Paris, France; 8 Inserm U891, Centre de Recherche en Cancérologie de Marseille, Molecular and Functional Hematopoiesis, Centre de référence des mastocytoses, 27 Bd Leï roure, 13009 Marseille, France; 9 Institut Paoli-Calmettes, Marseille, France; Université Méditerranée, 27 Bd Leï roure, 13009 Marseille, France; 10 CNRS UMR 8147, Service d'hématologie et centre de référence des mastocytoses, Hôpital Necker, 149 Rue de Sèvres, 75743 Paris, France; 11 Service de Rhumatologie, Hôpital de Hautepierre, Avenue Molière – BP 49 – 67098 Strasbourg, France

## Abstract

**Introduction:**

Since current treatment options for patients suffering from active rheumatoid arthritis (RA) remain inadequate, especially for those unresponsive to disease-modifying antirheumatic drugs (DMARDs), new and improved medication is needed. This study evaluates the safety and efficacy of masitinib (AB1010), a potent and selective protein tyrosine kinase inhibitor of c-KIT, in the monotherapy treatment of DMARD-refractory RA.

**Methods:**

This was a multicentre, uncontrolled, open-label, randomised, dose-ranging, phase 2a trial. Masitinib was administered orally to 43 patients who had inadequate response to DMARDs, at initial randomised dosing levels of 3 and 6 mg/kg per day over a 12-week period. Dose adjustment was permitted based upon tolerability and response criteria. Efficacy was assessed via American College of Rheumatology 20%/50%/70% improvement criteria (ACR20/50/70) responses, disease activity score using 28 joint counts (DAS28), index of improvement in RA (ACRn) and C-reactive protein (CRP) improvement, relative to baseline at week 12.

**Results:**

Improvement was observed in all efficacy endpoints, including ACR20/50/70 scores of 54%, 26% and 8%, respectively, and a reduction in CRP level by greater than 50% for approximately half the population. This improvement was sustainable throughout an extension phase (> 84 weeks) and was also independent of initial DMARD resistance (anti-tumour necrosis factor-alpha and/or methotrexate). A relatively high patient withdrawal rate (37%) required the use of last observation carried forward (LOCF) data imputation. Incidence of adverse events was high (95%), although the majority were of mild or moderate severity with a considerable decline in frequency observed after 12 weeks of treatment. Two nonfatal serious adverse events were reported. Dose-response analyses tentatively indicate that an initial dosing level of 6.0 mg/kg per day administered orally in two daily intakes is the most appropriate, based upon potency and tolerability trends.

**Conclusions:**

Treatment with masitinib improved DMARD-refractory active RA. Following an initial high incidence of mostly mild to moderate side effects during the first 12 weeks of treatment, masitinib appears to be generally well tolerated. This, together with evidence of a sustainable efficacy response, suggests that masitinib is suitable for long-term treatment regimens. Since this was the first study of masitinib in a nononcologic pathology, the relatively high patient withdrawal rate observed can be partly attributed to a highly cautious response to adverse events. There is sufficient compelling evidence to warrant further placebo-controlled investigation.

**Trial registration:**

ClinicalTrials.gov NCT00831922.

## Introduction

Rheumatoid arthritis (RA) has a complex aetiopathogenesis necessitating that a patient's treatment be individually and continually tailored for effective management. Disease-modifying antirheumatic drugs (DMARDs), especially methotrexate (MTX), have become the cornerstone of RA treatment. A shortcoming of MTX, however, is that it is relatively ineffective at inducing remission, with disease progression continuing unabated in many patients [1,2]. A problem more general to DMARDs is that of drug resistance, which represents a major obstacle to the effective long-term management of RA [3]. Both MTX [4] and anti-tumour necrosis factor-alpha (anti-TNFα) [5] may become inefficient for controlling disease activity in severe RA. Thus, beyond the already developed biological strategies, there exists an imperative need to identify alternative RA treatments that demonstrate high efficacy over time in monotherapy, exploit novel therapeutic targets for more effective combination therapies, minimise toxicity and are affordable. One such approach involves blocking intracellular proinflammatory messages, which is currently represented by the strategy of selective protein tyrosine kinase (TK) inhibition.

There is a growing body of evidence implicating mast cells (MCs) as major contributors to the pathogenesis of RA. MCs may be considered the immunological sentinel of the synovium, acting immediately in the event of joint trauma by liberating an array of proinflammatory mediators. However, MCs also appear to perpetuate the chronic process by their marked increased accumulation in the synovial lining of the inflamed joint and their ability to produce numerous proinflammatory cytokines and growth and angiogenic factors. Some of the most compelling evidence for the connection of MCs to RA comes from studies in the K/BxN murine model, an animal model of autoantibody-induced arthritis, which has demonstrated that MC-deficient mice are resistant to arthritis, with susceptibility restored following MC engraftment [6]. This model has also been used to show how MCs contribute to the initiation of joint inflammation by elaboration of interleukin-1 (IL1) [7]. As such, MCs represent an attractive therapeutic target [8-13]. Stem cell factor (SCF), the ligand of the c-KIT receptor, is a critical growth factor for MCs and is essential to their survival, proliferation, differentiation, adhesion and degranulation processes [14]. Thus, there exists a strong relation between the SCF/MC c-KIT pathway and the pathogenesis of RA. It is hypothesised that, if this link were disrupted through the inhibitory action of c-KIT TK activity, then inflammatory diseases such as RA could be controlled; that is, MCs are strongly implicated in RA pathogenesis, SCF is closely associated with MCs, and c-KIT is intrinsically linked with SCF; hence, inhibition of the c-KIT pathway affects RA. Small molecules capable of blocking ATP binding and TK activity of c-KIT, both selectively and with a good safety profile, could therefore represent a new class of drugs effective in RA.

Masitinib (AB1010), the investigatory drug of this study, is a good candidate, being an ATP-binding site competitor that acts potently and selectively by inhibiting wild-type forms of c-KIT. *In vitro *masitinib has shown greater affinity and selectivity for human and murine c-KIT receptor (wild-type: half inhibitory concentration [IC_50_] of 150 nM; juxtamembrane mutation: IC_50 _of 5 nM; P Dubreuil, S Letard, MA Ciufolini, L Gros, PS Leventhal, M Humbert, N Castéran, L Borge, B Hajem, A Lermet, W Sippl, E Voisset, M Arock, C Auclair, PS Leventhal, CD Mansfield, A Moussy & O Hermine, manuscript submitted) as compared with imatinib mesylate (Gleevec, STI571; Novartis, Basel, Switzerland), the forerunner of such therapeutic agents. Masitinib also potently inhibits platelet-derived growth factor receptor-alpha (PDGFRα), PDGFRβ, Lyn and (to a lesser extent) fibroblast growth factor receptor 3 (FGFR3) and the focal adhesion kinase (FAK) activation pathway without inhibiting kinases of known toxicities (P. Dubreuil and colleagues, manuscript submitted). The maximal tolerated dose of masitinib has not been reached thus far in phase 1 studies of healthy volunteers or in cancer patients who were orally administered up to 1,000 mg/day (corresponding to a weight-adjusted dose of not more than 20 mg/kg per day for patients weighing at least 50 kg; JC Soria, C Massard, N Magné, CD Mansfield, T Bader, A Moussy, O Hermine & JP Armand, manuscript in preparation). However, it was observed that doses of higher than 12 mg/kg per day lead to gastrointestinal disorders that are probably not compatible with a long-term administration of masitinib. Dose levels of 7.5 mg/kg per day have shown no significant toxicity, with plasmatic concentrations of masitinib base detected at levels above the IC_50 _for c-KIT and PDGFR (J.C. Soria and colleagues, manuscript in preparation). The purpose of this current study was to evaluate the safety and efficacy of masitinib in the treatment of DMARD-refractory active RA.

## Materials and methods

### Patients

Patients from 18 to 75 years of age who had been diagnosed with active RA, according to the American College of Rheumatology (ACR) criteria [15], for whom disease onset had occurred after 16 years of age and who had a history of DMARD failure (predominantly MTX and/or anti-TNFα) or primary resistance to anti-TNFα were eligible to participate. Their active RA had an ACR functional class of 1 to 3 [16] and a duration of at least 6 months. In addition, patients exhibited at least 8/66 swollen joints, at least 10/68 painful joints and at least one of the following three conditions: erythrocyte sedimentation rate of at least 28 mm/hour, C-reactive protein (CRP) of at least 15 mg/litre or morning stiffness for at least 45 minutes at both screening and baseline time points. The main exclusion criteria were patients with inadequate bone marrow function (defined as an absolute neutrophil count of not more than 2.5 × 10^9^/litre) and a platelet count of not more than 100 × 10^9^/litre, active current infection, history of infection requiring hospitalisation, history of recurrent infections or treatment with antibiotics within 2 weeks of screening. Treatment washout or exclusion periods observed prior to entry to the study were (a) DMARD use within 4 weeks, (b) five half-lives or washout in accordance with a specific drug (whichever is longer) (c) any live (attenuated) vaccines taken within 4 weeks, (d) use of more than one nonsteroidal anti-inflammatory drug (NSAID) or change of its dosage within 4 weeks, (e) dosage of prednisone or equivalent corticosteroid of greater than 10 mg/day or any dosage change within 4 weeks, and (f) dosage of prednisone or equivalent corticosteroid of greater than 20 mg administered via intra-articular injection or bolus intramuscular or intravenous treatment within 4 weeks. Other exclusion criteria included any previous use of recombinant IL1 receptor antagonist (IL1-Ra) and patients who were pregnant or nursing.

### Study design and drug product

This was a multicentre, prospective, uncontrolled, open-label, randomised, dose-ranging, phase 2a study of masitinib in adults with active RA, who were followed over the course of a 12-week period. The study was approved by the local ethics committees and was carried out in compliance with the Declaration of Helsinki and good clinical practices guidelines. Written informed consent was obtained from all patients. The study was registered in ClinicalTrials.gov under the trial registration number NCT00831922.

Masitinib, supplied as 100 and 200 mg tablets (AB Science, Paris, France), was administered orally in two daily intakes. To evaluate the dose response of masitinib in DMARD-refractory active RA, dose ranging was performed by randomly assigning patients to one of two initial treatment groups of 3 and 6 mg/kg per day (1:1 ratio). Dosage could be increased by 1.5 mg/kg per day at weeks 4 and 8 in the event of insufficient response accompanied by minimal toxicity. Likewise, the dose could be reduced by 1.5 mg/kg per day or treatment discontinued in case of serious adverse events (SAEs). Patients exhibiting a significant improvement after 12 weeks of treatment were eligible to continue receiving treatment after entering a compassionate program, wherein assessments were performed every 4 weeks for the first 3 months of extension and every 12 weeks thereafter.

Permitted medications for the treatment of possible cutaneous rash and face oedema during the study were hydroxyzine (Atarax) and prednisolone. Other permitted concomitant medications were one NSAID (including cyclooxygenase 2 [COX-2] inhibitors) at constant dosage, oral corticosteroids at stable doses of not more than 10 mg/day, analgesics without anti-inflammatory action or oral narcotic analgesics and medically acceptable forms of birth control. Physical therapy, if performed at the time of study entry, was provided under a stable and consistent regimen. The following treatments of active RA were prohibited during the study: surgery, DMARD treatment (including MTX, anti-TNFα biology therapies, leflunomide, IL1-Ra, azathioprine and cyclosporine), immunosuppressive drugs, cytotoxic drugs, intramuscular or intravenous injections of steroids, intra-articular or soft tissue injections of corticosteroids and alternate investigational drugs or investigational combinations of approved drugs. Drugs that interact with the same CYP450 isoenzymes (2C9, 2D6 and 3A4) as masitinib were prohibited (for example, acetaminophen) due to the inherent risk of either reduced activity or enhanced toxicity of any concomitant medication. Finally, the use of analgesics was prohibited on assessment days until after all clinical efficacy evaluations had been completed.

### Safety and efficacy assessment

Safety was assessed by occurrence of adverse events (AEs) and SAEs and monitoring biochemical, haematological and urinalysis parameters during the study period, with toxicity graded according to the Common Toxicity Criteria version 3.0. In the event of SAE (that is, grade 3 or 4), treatment was interrupted until resolution and then resumed, with a permitted dose reduction of 1.5 mg/kg per day or treatment discontinuation if toxicity recurred. Evaluation of treatment efficacy was based upon the evolution of clinical symptoms associated with active RA at week 12 relative to baseline. Primary endpoints were the ACR response criteria of ACR20, ACR50 and ACR70 [17]. For each patient, all efficacy parameters were recorded on the first day of treatment (baseline), prior to administration of masitinib and then again after 4, 8 and 12 weeks of treatment. Secondary endpoints included the 12-week analysis of disease activity score using 28 joint counts (DAS28) [18], index of improvement in RA (ACRn) [19] and CRP improvement. Higher DAS28 values are indicative of greater disease activity with significance placed on the threshold values of DAS28 < 2.6, 2.6 ≤ DAS28 ≤ 3.2, 3.2 < DAS28 ≤ 5.1, and DAS28 > 5.1, corresponding to the classifications of remission, inactive RA, moderate RA and very active RA, respectively. CRP is an acute-phase reactant and a sensitive serum marker of inflammation. Discrimination between dose regimens was investigated by analysis of the time (days) to first ACR variable response according to initial dosage. Since dose adjustment was permitted at weeks 4 and 8 in cases of insufficient treatment response, the dose at the time of first response was also analysed.

### Statistical methods

Efficacy data are presented using descriptive statistics, contrasting initial dosage groups or according to previous DMARD failure. For comparison of groups according to initial dosage on a continuous variable, the Student test (with Satterwhaite correction for unequal variance) or the Wilcoxon test was used when normality was not rejected or was rejected, respectively (normality determined via the Shapiro-Wilk test). For the same comparison on a qualitative variable, the chi-square or Fisher exact test (if the chi-square hypotheses were not fulfilled) was used. The rates of patients achieving the various ACR response variables after 12 weeks of treatment (remission rate) are presented in terms of number and percentage of patients. Patients were assigned to either 3 or 6 mg/kg per day treatment groups based upon a randomisation schedule generated for packaging and labelling by the Biostatistics Section of AB Science. Individual treatment doses to be administered were supplied in sealed envelopes to be opened by the investigator at the time of inclusion. Patients received the treatment from the investigator on an open basis.

Due to the relatively high patient dropout rate of this study, analysis was conducted on two different datasets: one with an imputation of missing values according to the last observation carried forward (LOCF) methodology and the other in the absence of data imputation (that is, the observed cases [OCs]). Analysis for efficacy was performed on a modified intention-to-treat (ITT) population and per protocol (PP) population. The ITT population was defined as those patients who had received at least one dose of masitinib and who had undergone at least one post-baseline assessment of efficacy. The PP population was defined as a subgroup of the ITT population that in addition had presented no major protocol deviations and had completed at least 28 days of treatment exposure.

## Results

### Baseline characteristics and participant flow

Between December 2004 and March 2006, a total of 43 patients were enrolled in the study. Participants were randomly assigned to one of two initial treatment groups, receiving a masitinib dosage of either 3 mg/kg per day (n = 22) or 6 mg/kg per day (n = 21). Of these, 27/43 (63%) patients completed the study, with 21/43 (49%) patients entering the study's extension phase (10/43 [23%] patients received treatment for more than 1 year, 8/43 [19%] for more than 2 years and 3/43 [7%] for more than 3 years). Of the 16 (37%) patients who withdrew before completion of the 12-week study period, occurrence of an AE was cited as the primary cause of discontinuation. Participant baseline characteristics, disposition and dosing history are presented in Table 1 according to the randomised dose-ranging treatment groups. Baseline values of several efficacy parameters were higher in the 6 mg/kg per day group compared with the 3 mg/kg per day group; for example, DAS28 was, respectively, 7.1 versus 6.1 (*P *= 0.010), CRP was 62 versus 26 mg/litre (*P *= 0.029), swollen joint count was 22.1 versus 15.3 (*P *= 0.046), previous anti-TNFα was 67% versus 36% (*P *= 0.056) and Health Assessment Questionnaire score was 2.2 versus 1.9 (*P *= 0.082). Hence, the 6 mg/kg per day initial dosage arm had a higher baseline of disease severity.

**Table 1:** Baseline characteristics, overall disposition and dosing history, according to initial dosage

Parameter	Masitinib 3 mg/kg per day (n = 22)	Masitinib 6 mg/kg per day (n = 18)	Total population (n = 40)
Demographic (intent-to-treat population)			
Age, years			
Mean ± SD	54.0 ± 12.2	55.5 ± 9.2	54.7 ± 10.8
Range	27.0–75.0	34.0–69.0	27.0–75.0
Weight, kg			
Mean ± SD	67.1 ± 12.8	69.2 ± 20.5	68.1 ± 16.5
Range	49.0–88.0	50.0–136.0	49.0–136.0
Gender			
Male	3/22 (13.6%)	6/18 (33.3%)	9/40 (22.5%)
Female	19/22 (86.4%)	12/18 (66.7%)	31/40 (77.5%)
			
Clinical (intent-to-treat population)			
Disease duration in years, mean ± SD	11.8 ± 5.9	10.7 ± 8.1	11.3 ± 6.9
Tender joints, mean ± SD	24.7 ± 11.1	32.2 ± 16.3	28.1 ± 14.0
Swollen joints, mean ± SD	15.3 ± 10.4	22.1 ± 12.0	18.4 ± 11.5
Patient pain assessment, mean ± SD	67.4 ± 19.2	68.6 ± 27.4	67.9 ± 23.0
Patient assessment of DA, mean ± SD	69.4 ± 24.9	73.0 ± 22.9	71.0 ± 23.8
Physician assessment of DA, mean ± SD	66.4 ± 19.5	66.8 ± 18.8	66.6 ± 18.9
HAQ score, mean ± SD	1.9 ± 0.6	2.2 ± 0.5	2.0 ± 0.6
CRP (mg/litre), mean ± SD	26.2 ± 28.4	62.3 ± 57.6	42.3 ± 46.9
DAS28, mean ± SD	6.1 ± 0.8	7.1 ± 1.1	6.5 ± 1.0
DMARD failures (percentage)			
Anti-TNFα	8/22 (36.4%)	12/18 (66.7%)	20/40 (50.0%)
Other	14/22 (63.6%)	6/18 (33.3%)	20/40 (50.0%)
			
Patient disposition (randomised population)			
	Masitinib 3 mg/kg per day (n = 22)	Masitinib 6 mg/kg per day (n = 21)	Total population (n = 43)
Early study discontinuation	7/22 (31.8%)	9/21 (42.9%)	16/43 (37.2%)
Insufficient therapeutic effect	1/7 (14.3%)	1/9 (11.1%)	2/16 (12.5%)
Protocol violation	0/7 (0.0%)	0/9 (0.0%)	0/16 (0.0%)
Adverse event	6/7 (85.7%)	7/9 (77.8%)	13/16 (81.3%)
Consent withdrawn	0/7 (0.0%)	1/9 (11.1%)	1/16 (6.3%)
End of study without extension	5/22 (22.7%)	1/21 (4.8%)	6/43 (14.0%)
Entered extension phase	10/22 (45.4%)	11/21 (52.3%)	21/43 (48.9%)
			
Dosing adjustment (intent-to-treat population over 12-week study phase)			
	Masitinib 3 mg/kg per day (n = 22)	Masitinib 6 mg/kg per day (n = 18)	Total population (n = 40)
No dose adjustment	10/22 (45%)	8/18 (44%)	18/40 (45%)
Increase by 1.5 mg/kg per day	6/22 (27%)	3/18 (17%)	9/40 (23%)
Increase by 3.0 mg/kg per day	2/22 (9%)	5/18 (28%)	7/40 (18%)
Increase by 4.5 mg/kg per day	3/22 (14%)	0/18 (0%)	3/40 (8%)
Other^a^	1/22 (5%)	2/18 (11%)	3/40 (8%)

Active rheumatoid arthritis patients were randomly assigned to receive masitinib therapy at initial dosing levels of 3.0 or 6.0 mg/kg per day, administered *per os *for 12 weeks. Dose adjustment was permitted depending upon efficacy and safety assessments. Pain and disease activity were assessed using an EQ-5D (EuroQoL-5 Dimensions) visual analogue scale. ^a^Combination of dose augmentation and/or diminution. Anti-TNFα, anti-tumour necrosis factor-alpha; CRP, C-reactive protein; DA, disease activity; DAS28, disease activity score using 28 joint counts; DMARD, disease-modifying antirheumatic drug; HAQ, Health Assessment Questionnaire; SD, standard deviation.

Three patients were excluded from the randomised population due to lack of efficacy data following baseline; thus, according to our ITT population definition, the resulting ITT population was n = 40. This corresponded to 3 and 6 mg/kg per day randomised dose-ranging groups of n = 22 and n = 18, respectively. Four other patients were excluded from the PP population (n = 36 with n = 18 for each group): one due to a major protocol violation (that is, treated with prednisone at 20 mg/day before baseline) and three due to insufficient exposure time (that is, fewer than 28 days).

In regard to analysis of the primary efficacy outcome (that is, ACR score at week 12), 39/40 (97%) patients had sufficient post-baseline data available for analysis in the ITT LOCF group. (The size of this efficacy analysis group differs from that of the ITT population since, although the missing patient fulfilled the ITT criteria, he did not possess a sufficiently complete dataset to permit calculation of the multiparametric ACR score.) The PP OC efficacy analysis group had sufficient data available for analysis of 27/36 (75%) patients. Secondary efficacy outcomes were likewise analysed according to the number of patients possessing sufficient data for evaluation at 12 weeks.

Subgroup analysis of the ITT population with respect to previous DMARD treatment failure revealed that 20/40 (50%) patients were unresponsive to anti-TNFα (including 5/40 [12%] patients resistant to one anti-TNFα, 10/40 [25%] patients resistant to more than one anti-TNFα and 5/40 [12%] patients intolerant to anti-TNFα). In addition, 33/40 (82%) patients were unresponsive to MTX. Among them, 18 patients were unresponsive to both anti-TNFα and MTX. Analyses of the participant baseline characteristics with respect to previous treatment failure (data not shown) suggest that, although the entire population was classified as having 'very active RA', those patients previously treated with anti-TNFα were suffering from RA of even greater severity than that of the other patients.

### Safety and tolerability of masitinib

Assessment of safety was performed on all patients who had received at least one dose of masitinib (n = 43) over the study duration, including the treatment extension period with a cutoff date of 31 August 2008. Overall patient exposure to masitinib was 288 ± 378 days on average, with a median exposure of 91 days and a range of 8 to 1,274 days. The incidence of common (> 4%) treatment-related AEs according to intensity is presented in Table 2 for the initial (12-week study period) and extension phases. A total of 40/43 (93%) patients reported at least one masitinib-related (or not assessable) AE during the initial phase. In general, AEs were transient in nature and of mild to moderate intensity; nevertheless, occurrence of AEs was the main reason that 13/43 (30%) patients discontinued treatment. In 9/43 (21%) patients, the AEs were severe, including oedema and rash in 3/43 (7%) and 2/43 (5%) patients, respectively. One patient presented with angioedema of moderate intensity (face oedema, rash and dyspnea without hypotension or any sign of shock). This event resolved upon masitinib interruption and without specific medications, ruling out any anaphylactic or anaphylactic-like reaction. No changes considered to be of clinical relevance were observed in regard to physical, haematological or urinalysis parameters during the initial phase; however, 1/43 (2%) patient presented with hepatic disorder of increased liver enzymes (aspartate amino transferase: 122 units/litre, alanine amino transferase: 188 units/litre and alkaline phosphatase: 635 units/litre) at a dose of 6 mg/kg per day. This episode, reported as a severe transaminase increase AE, occurred after 14 days of treatment and resolved within 4 weeks of drug withdrawal, with no reoccurrence following the reintroduction of treatment. Analysis of AEs with respect to the dose of their occurrence (data not shown) showed that no clear dose-toxicity relationships exist, with the exception of oedema. The number of patients experiencing at least one oedema was 11/43 (26%), with 6/36 (16.7%) for doses of not more than 6.0 mg/kg per day and 5/15 (33.3%) for doses of greater than 6.0 mg/kg per day. Such oedematous episodes typically occurred 4 weeks (median onset time of 28 days) after the first drug intake or dose increase and abated within an average of 16 days. Four (9%) patients reported nonfatal SAEs of severe intensity which were suspected to be related to masitinib (or not assessable) and which consisted of skin rash, pleural effusion, pneumonia and RA flare-up. Only one of those SAEs (pleural effusion) resulted in patient withdrawal. All of these patients recovered without sequelae, and no deaths occurred during this study.

**Table 2:** Number (percentage) of subjects with at least one suspected (or not assessable) adverse event, according to intensity

Initial phase				
System organ class/preferred term^a^	All (n = 43)	Mild	Moderate	Severe
At least one suspected AE^b^	40 (93.0%)	29 (67.4%)	27 (62.8%)	9 (20.9%)
Rash-All categories	13 (30.2%)	7 (16.3%)	8 (18.6%)	2 (4.7%)
Oedema-All categories	11 (25.6%)	2 (4.7%)	6 (14.0%)	3 (7.0%)
Nausea	10 (23.3%)	6 (14.0%)	5 (11.6%)	
Diarrhoea	8 (18.6%)	5 (11.6%)	2 (4.7%)	1 (2.3%)
Headache	6 (14.0%)	4 (9.3%)	2 (4.7%)	
Abdominal pain, upper	5 (11.6%)	4 (9.3%)	1 (2.3%)	
Vomiting	5 (11.6%)	1 (2.3%)	4 (9.3%)	
Asthenia	5 (11.6%)		4 (9.3%)	1 (2.3%)
Pyrexia	3 (7.0%)	1 (2.3%)	1 (2.3%)	1 (2.3%)
Herpes simplex	3 (7.0%)	2 (4.7%)	1 (2.3%)	
Weight decreased	3 (7.0%)	2 (4.7%)	1 (2.3%)	
Dyspnoea	3 (7.0%)	1 (2.3%)	1 (2.3%)	1 (2.3%)
Abdominal pain	2 (4.7%)	1 (2.3%)		1 (2.3%)
Dry mouth	2 (4.7%)	1 (2.3%)		1 (2.3%)
Hyperthermia	2 (4.7%)		1 (2.3%)	1 (2.3%)
Gastroenteritis	2 (4.7%)		2 (4.7%)	
Blood creatinine increased	2 (4.7%)	1 (2.3%)	1 (2.3%)	
Cough	2 (4.7%)	1 (2.3%)	1 (2.3%)	
Alopecia	2 (4.7%)	2 (4.7%)		
Petechiae	2 (4.7%)	1 (2.3%)	1 (2.3%)	
				
Extension phase				

System organ class/preferred term	All (n = 21)	Mild	Moderate	Severe
At least one suspected AE	10 (47.6%)	4 (19.0%)	3 (14.3%)	3 (14.3%)
Oedema-All categories	2 (9.5%)		2 (9.5%)	
Leukopenia	1 (4.8%)		1 (4.8%)	
Vertigo	1 (4.8%)		1 (4.8%)	
Aphthous stomatitis	1 (4.8%)	1 (4.8%)		
Asthenia	1 (4.8%)	1 (4.8%)		
Pyrexia	1 (4.8%)	1 (4.8%)		
Liver disorder	1 (4.8%)			1 (4.8%)
Gastroenteritis	1 (4.8%)	1 (4.8%)		
Nasopharyngitis	1 (4.8%)	1 (4.8%)		
Rhinitis	1 (4.8%)	1 (4.8%)		
Neutrophil count decreased	1 (4.8%)	1 (4.8%)		
Rheumatoid arthritis	1 (4.8%)			1 (4.8%)
Bronchopneumopathy	1 (4.8%)			1 (4.8%)
Pleural effusion	1 (4.8%)			1 (4.8%)
Eczema	1 (4.8%)	1 (4.8%)		
Onychoclasis	1 (4.8%)	1 (4.8%)		
Photosensitivity reaction	1 (4.8%)	1 (4.8%)		

Table includes those adverse events (AEs) that occurred commonly (that is, in greater than 4% of patients). ^a^MedDRA (medical dictionary for regulatory activities) terminology. ^b^AE intensity count is cumulative. AEs were recorded only once (at their start date).

For patients entering the extension phase (n = 21), a clear decrease in the occurrence of AEs as well as a reduction in severity were evident. Overall, 10/21 (48%) patients reported at least one masitinib-related (or not assessable) AE; these AEs were of mild, moderate or severe intensity in 4/21 (19%), 3/21 (14%) and 3/21 (14%) patients, respectively. Specifically, no incidence of skin rash, nausea, vomiting or diarrhoea was reported after week 12, and occurrence of oedema decreased more than 60%.

### Clinical efficacy of masitinib

Evaluation of the primary efficacy endpoint ACR and the secondary endpoints of ACRn, DAS28 and CRP improvement is presented in Table 3 according to the ITT LOCF and PP OC analysis groups. Treatment with masitinib significantly improved the severity of active RA: at week 12, ACR20, ACR50 and ACR70 were achieved by 15/27 (55.6%), 9/27 (33.3%) and 3/27 (11.1%) patients, respectively, in the PP OC group. The corresponding numbers in the ITT LOCF group were 21/39 (53.8%), 10/39 (25.6%) and 3/39 (7.7%). These results are presented as the cumulative number of patients reaching each ACR level, with performance observed to be similar between efficacy analysis groups; the slightly lower response in ITT LOCF was attributable to the fact that imputed data were typically associated with patient withdrawal and, therefore, a lower treatment exposure. Considerable improvement was also observed in the ACRn analysis, the PP OC and ITT LOCF analysis groups achieving an improvement of 31.6 and 23.0 units, respectively, at week 12. With respect to DAS28 values, the PP OC and ITT LOCF populations exhibited an absolute change of 2.0 and 1.7 units, respectively, from a baseline of 6.5 units, representing an improvement in DAS28 classification from 'very active RA' to 'moderate RA'. In regard to the number of patients with a DAS28 of less than 2.6 (classified as disease remission), two patients from the ITT LOCF population's MTX subgroup exhibited this improvement but none from the anti-TNFα subgroup did. Finally, approximately 50% of patients experienced a significant reduction (> 50%) in their CRP levels, signifying a decrease in their inflammation.

**Table 3:** Summary of efficacy outcomes at week 12 with subgroup analysis according to previous treatment failure

Parameter	PP OCs			ITT LOCF		
		
	All patients	Resistance to anti-TNFα	Resistance to MTX	All patients	Resistance to anti-TNFα	Resistance to MTX
ACR^a^	(n = 27)	(n = 14)	(n = 23)	(n = 39)	(n = 19)	(n = 32)
ACR20	15/27 (55.6%)	8/14 (57.1%)	14/23 (60.9%)	21/39 (53.8%)	10/19 (52.6%)	17/32 (53.1%)
ACR50	9/27 (33.3%)	4/14 (28.6%)	9/23 (39.1%)	10/39 (25.6%)	4/19 (21.1%)	9/32 (28.1%)
ACR70	3/27 (11.1%)	1/14 (7.1%)	3/23 (13.0%)	3/39 (7.7%)	1/19 (5.3%)	3/32 (9.4%)
						
ACRn						
Mean ± SD	31.6 ± 33.5	28.1 ± 32.1	36.6 ± 31.6	23.0 ± 37.5	18.7 ± 36.8	24.1 ± 38.8
Median	42.9	44.3	46.9	25.7	20.6	32.7
Range	-40.0–87.5	-40.0–72.2	-40.0–87.5	-62.5–87.5	-62.5–72.2	-62.5–87.5
						
CRP	(n = 28)	(n = 14)	(n = 23)	(n = 35)	(n = 17)	(n = 29)
Improvement > 50%	14/28 (50.0%)	7/14 (50.0%)	12/23 (52.2%)	19/35 (54.3%)	9/17 (52.9%)	16/29 (55.2%)
25% < improvement ≤ 50%	3/28 (10.7%)	1/14 (7.1%)	2/23 (8.7%)	4/35 (11.4%)	2/17 (11.8%)	3/29 (10.3%)
0% ≤ improvement ≤ 25%	5/28 (17.9%)	1/14 (7.1%)	3/23 (13.0%)	5/35 (14.3%)	1/17 (5.9%)	3/29 (10.3%)
Stability	3/28 (11%)	3/14 (21%)	3/23 (13%)	3/35 (9%)	3/17 (18%)	3/29 (10%)
Deterioration	3/28 (11%)	2/14 (14%)	3/23 (13%)	4/35 (11%)	2/17 (12%)	4/29 (14%)
						
DAS28	(n = 24)	(n = 13)	(n = 20)	(n = 34)	(n = 18)	(n = 28)
Mean ± SD	4.6 ± 1.3	5.1 ± 1.2	4.6 ± 1.4	4.8 ± 1.5	5.2 ± 1.1	4.8 ± 1.5
ΔDAS28	2.0	1.8	2.1	1.7	1.7	1.8
Range	0.5–7.0	3.3–7.0	0.5–7.0	0.5–7.0	3.3–7.0	0.5–7.0
DAS28 < 2.6	1/24 (4.2%)	0/28 (0%)	1/20 (5.0%)	2/34 (5.9%)	0/18 (0%)	2/28 (7.1%)
DAS28 ≤ 3.2	1/24 (4.2%)	0/28 (0%)	1/20 (5.0%)	2/34 (5.9%)	0/18 (0%)	2/28 (7.1%)

^a^Primary efficacy outcome. American College of Rheumatology (ACR) results are presented as the cumulative number of patients reaching each ACR level. Population sizes could vary with respect to an efficacy endpoint due to the fact that, for some patients, all efficacy data under treatment were missing (no data imputation was possible in this case). ACR20/50/70, American College of Rheumatology 20%/50%/70% improvement criteria; ACRn, index of improvement in rheumatoid arthritis; anti-TNFα, anti-tumour necrosis factor-alpha; CRP, C-reactive protein; DAS28, disease activity score using 28 joint counts; ΔDAS28, the change in disease activity score using 28 joint counts from baseline; ITT, intention-to-treat; LOCF, last observation carried forward; MTX, methotrexate; OC, observed case; PP, per protocol population; SD, standard deviation.

The pattern of masitinib efficacy appears to be independent of previous treatment failure, with approximately 50% of patients achieving the ARC20 and ΔCRP greater than 50% response criteria regardless of previous treatment (Table 3); that is, masitinib is equally effective in patients for whom previous treatment with anti-TNFα or MTX has been inadequate. Preliminary results from the extension phase are of major interest since they show the observed improvement to be consistently maintained over a duration of more than 84 weeks, demonstrating masitinib's sustainability (Table 4). In regard to the DAS28 extension phase data after 1 year of treatment (60 weeks), an increasing number of patients were achieving DAS28 values of not more than 3.2 or less than 2.6, signifying inactive RA or an increased likelihood of being in remission. Furthermore, over this time, two patients achieved up to 90% improvement (ACR90). Taken together, this suggests that further therapeutic gains could possibly be achieved given longer exposure times.

**Table 4:** Efficacy outcomes^a ^from the extension phase of the study: weeks 12 to 82 (intention-to-treat population)

Parameter	W12	W24	W36	W48	W60	W72	W84
ACR, number (percentage) of patients	n = 27	n = 7	n = 9	n = 8	n = 8	n = 9	n = 8
ACR20^b^	15 (56%)	6 (86%)	7 (78%)	5 (63%)	6 (75%)	6 (67%)	7 (88%)
ACR50^b^	9 (33%)	2 (27%)	4 (44%)	3 (38%)	6 (75%)	3 (33%)	5 (63%)
ACR70	3 (11%)	1 (14%)	2 (22%)	1 (13%)	3 (38%)	2 (22%)	2 (25%)
ACR90	0 (0%)	0 (0%)	1 (11%)	0 (0%)	2 (25%)	1 (11%)	1 (13%)
							
ACRn	n = 27	n = 7	n = 9	n = 8	n = 8	n = 9	n = 8
Mean ± SD	31.6 ± 33.5	36.0 ± 29.0	45.9 ± 32.3	30.9 ± 36.7	58.3 ± 31.4	35.6 ± 41.3	50.9 ± 38.0
Median	42.9	40.7	45.5	40.0	64.9	39.7	55.0
Range	-40.0–87.5	-16.7–73.0	-3.8–93.3	-20.0–70.9	10.0–93.3	-27.8–97.4	-17.6–98.8
							
CRP, number (percentage) of patients	n = 28	n = 7	n = 12	n = 9	n = 7	n = 9	n = 8
Improvement > 50%	14 (50%)	5 (71%)	9 (75%)	6 (67%)	3 (43%)	6 (68%)	5 (63%)
25% < improvement ≤ 50%	3 (11%)	0 (0%)	1 (8%)	0 (0%)	3 (43%)	1 (11%)	1 (13%)
0% < improvement ≤ 25%	5 (18%)	1 (14%)	1 (8%)	1 (11%)	1 (14%)	1 (11%)	1 (13%)
Stable	3 (11%)	1 (14%)	1 (8%)	0 (0%)	0 (0%)	0 (0%)	0 (0%)
Deterioration	3 (11%)	0 (0%)	0 (0%)	2 (22%)	0 (0%)	1 (11%)	1 (13%)
							
DAS28	n = 24	n = 4	n = 5	n = 6	n = 7	n = 7	n = 4
Mean ± SD	4.6 ± 1.3	5.2 ± 1.7	4.4 ± 1.9	4.7 ± 2.1	3.3 ± 1.5	3.5 ± 1.5	3.1 ± 1.6
Median	4.4	4.9	4.1	4.4	2.6	3.0	2.5
Range	0.5–7.0	3.6–7.5	2.3–7.5	2.7–8.7	1.7–5.3	1.6–6.1	1.9–5.5
DAS28 < 2.6, number (percentage) of patients	1 (4%)	0 (0%)	1 (20%)	0 (0%)	4 (57%)	1 (14%)	2 (50%)
DAS28 ≤ 3.2, number (percentage) of patients	1 (4%)	0 (0%)	1 (20%)	2 (33%)	4 (57%)	4 (57%)	3 (75%)

^a^Results from extension phase are preliminary. ^b^Primary efficacy outcome. American College of Rheumatology (ACR) results are presented as the cumulative number of patients reaching each ACR level. ACR20/50/70/90, American College of Rheumatology 20%/50%/70%/90% improvement criteria; ACRn, index of improvement in rheumatoid arthritis; DAS28, disease activity score using 28 joint counts; CRP, C-reactive protein; SD, standard deviation; W, week.

### Dose analysis

An analysis of time to first response according to initial dosage is presented in Table 5. This analysis extends to the extension phase for a total assessment period of approximately 32 weeks. Patients randomly assigned to the 6 mg/kg per day dosing group achieved a response faster than those assigned to the 3 mg/kg per day (ACR20: median of 29 versus 56 days [*P *= 0.231]; ACR50: 72.5 versus 84 days [*P *= 0.771], respectively); however, these differences were not statistically significant (*P *< 0.05). In cases of insufficient treatment response, dose adjustment was permitted at weeks 4 and 8; hence, the dose at time of first response was also analysed. Results reveal that approximately 65% and 73% of those patients achieving ACR20 or ACR50 scores, respectively, did so at a dosage of not more than 6 mg/kg per day. Moreover, this dosage corresponded to the highest response rate (5/15, 33.3%) for the ACR50 threshold. For those patients randomly assigned to the 3 mg/kg per day dosing group, 12/22 (55%) received dose augmentation at weeks 4 or 8 due to insufficient response. Of these, 7/12 (58%) patients experienced an improved response within the initial 12-week phase whereas 5/12 (42%) patients were nonresponders, having failed to reach the ACR20 threshold.

**Table 5:** Time to first response (days) in intention-to-treat population, according to initial masitinib dosage

Parameter	3 mg/kg per day (n = 22)	6 mg/kg per day (n = 18)	Total population (n = 40)	*P *value
ACR20				
Patients	12/22 (55.0%)	11/18 (61.0%)	23/40 (57.5%)	0.213
Mean ± SD	51.9 ± 24.5	40.3 ± 19.0	46.3 ± 22.4	
Median	56.0	29.0	35.0	
Range	28.0–105.0	28.0–86.0	28.0–105.0	
ACR50				
Patients	7/22 (32.0%)	8/18 (44.0%)	15/40 (37.5%)	0.771
Mean ± SD	91.9 ± 59.5	86.8 ± 61.1	89.1 ± 58.2	
Median	84.0	72.5	84.0	
Range	28.0–217.0	28.0–203.0	28.0–217.0	

ACR20, American College of Rheumatology 20% improvement criteria; ACR50, American College of Rheumatology 50% improvement criteria; SD, standard deviation.

## Discussion

Although the incidence of AEs was high in the study population as a whole (95%), the majority of these were mild or moderate in severity, transitory in nature and resolved spontaneously or upon temporary treatment interruption. Moreover, because this was the first study of masitinib as treatment in a nononcologic pathology, the increased incidence of dermatological events typically associated with this therapeutic class [20] was understandably treated with great caution by patients and investigators alike. This may in part explain the relatively high dropout rate of patients. Of those who withdrew from the study because of AEs prior to week 12 (n = 13), 9/13 (69%) patients had experienced AEs of a mild or moderate intensity, which could feasibly have been managed without permanent interruption of treatment. In general, AEs occurred early during the course of treatment, which is consistent with the known safety profile of TK inhibitors [21]. This trend is clearly evident when comparing safety data from the initial and extension phases, the implication being that, although masitinib is not completely free from side effects, the majority of these are over following 12 weeks of treatment, with good tolerance experienced thereafter during any long-term treatment regimen. During the initial 12 weeks, the most common AEs were rashes, oedema, nausea and diarrhoea. Cutaneous rash may potentially be linked to the action of masitinib on MCs, inducing MC apoptosis with a subsequent release of various mediators (for example, histamine, prostaglandins or cytokines) that are responsible for rash. This apoptosis seems to happen only once. The time necessary for the released mediators to reach the reaction site and accumulate to a certain concentration in the skin might explain why such events typically manifest themselves between the second and third weeks of treatment. Diarrhoea may also be linked to the pharmacological activity of masitinib on MCs in the intestine or through direct action on Cajals cells of the intestine, which also express the c-KIT receptor. Oedema, mainly palpebral and face oedema, is thought to be linked to the activity of masitinib on PDGFR, a TK receptor involved in the vasculatory pressure of tissues, especially in the periorbital region sensible to low pressure.

Overall, the safety profile of masitinib for long-term treatment would appear favourable, especially when considering concerns of cardiotoxicity and genotoxicity. For example, imatinib mesylate (Gleevec, STI571; Novartis, Basel, Switzerland) is cardiotoxic due to its strong inhibition of the Abelson kinase (ABL) [22,23], making its long-term use questionable for treatment of active RA. Masitinib, in contrast, is a weak inhibitor of BCR-ABL (and is also either an inactive or a weak inhibitor of other known cardiotoxic kinases such as Src and vascular endothelial growth factor receptor [VEGFR]), implying that masitinib may exhibit a better safety profile than other TK inhibitors, particularly on cardiac functions (P. Dubreuil and colleagues, manuscript submitted). Preclinical studies have also shown that masitinib is not genotoxic.

The performance of masitinib, with respect to the primary endpoint ACR scores, compares favourably to other biological DMARDs, including rituximab, abatacept and adalimumab [24-27]. Moreover, due to a lack of dosage increase in the event of insufficient response without toxicity (a protocol deviation), some patients may not have benefited from an optimal masitinib dose with a consequent reduction in efficacy results. Observed clinical improvement was supported by laboratory evidence of reduced inflammation in the form of a significant and sustainable decrease in CRP level for approximately half the study population. This result is important since, in the absence of a control group, it serves as proof that the observed improvements are attributable to the treatment. The results from other secondary endpoints (ACRn and DAS28) provide additional evidence of efficacy, with consistent patterns to the primary endpoint regarding sustainability and independence from previous treatment failure.

Dose-response analyses tentatively indicate that a dose level of 6 mg/kg per day is the most potent, although inequality of baseline clinical parameters between dose groups may be a confounding influence. Hence, no definite conclusion on the optimal initial dosing level can be reached. In regard to tolerability, the majority of severe AEs were associated with doses of at least 7.5 mg/kg per day. Thus, utilisation of not more than 6 mg/kg per day would likely reduce the occurrence of severe AEs, in particular those associated with oedema.

## Conclusions

Within the limitations of an uncontrolled phase 2a trial, this study has indicated that masitinib is a generally well-tolerated (especially after the initial 12 weeks) and effective treatment for DMARD-refractory active RA. Given the selective antimastocyte mechanism of action of masitinib, the results of this study help to further establish the critical role of MCs in the pathogenesis of active RA. More specifically, this study supports the viability of exploiting the SCF/c-KIT pathway as a therapeutic target. There is sufficient compelling evidence to proceed to phase 2b/3 randomised clinical trials to confirm and further characterise these findings.

## Abbreviations

ABL: Abelson kinase; ACR: American College of Rheumatology; ACR20/50/70/90: American College of Rheumatology 20%/50%/70%/90% improvement criteria; ACRn: index of improvement in rheumatoid arthritis; AE: adverse event; anti-TNFα: anti-tumour necrosis factor-alpha; CRP: C-reactive protein; DAS28: disease activity score using 28 joint counts; DMARD: disease-modifying antirheumatic drug; IC_50_: half inhibitory concentration; IL1: interleukin-1; IL1-Ra: (recombinant) interleukin-1 receptor antagonist; ITT: intention-to-treat; LOCF: last observation carried forward; MC: mast cell; MTX: methotrexate; NSAID: nonsteroidal anti-inflammatory drug; OC: observed case; PDGFR: platelet-derived growth factor receptor; PP: per protocol; RA: rheumatoid arthritis; SAE: serious adverse event; SCF: stem cell factor; TK: tyrosine kinase.

## Competing interests

AM, LG, PD and OH are employees and shareholders of the study sponsor, AB Science. PGi and CDM are employees of the study sponsor. AB Science is the proprietary holder of masitinib (AB1010). The other authors declare that they have no competing interests.

## Authors' contributions

JT was the main contributor to recruitment and treatment of the patients, contributing also to data analysis and interpretation and to preparation of the manuscript. XM was a co-coordinating clinical investigator who contributed to the study's conception and design, patient recruitment and treatment, data analysis and interpretation, and manuscript preparation. PB, R-MF, PGa and XLL were clinical investigators who contributed to the recruitment and treatment of the patients. PGi, AM and OH contributed to the study's conception and design, data analysis and interpretation, and manuscript preparation. LG contributed to the study's conception and design and to analysis and interpretation of the data. CDM, a medical writer at AB Science, contributed to data analysis and interpretation and was the main contributor in the preparation of the manuscript. PD contributed to the conception and design of the study and contributed analytical tools. JS was a co-coordinating clinical investigator who contributed to the study's conception and design, patient recruitment and treatment, and data analysis and interpretation. All authors critically reviewed the manuscript and gave final approval of the version to be published.
